# P268: One-day prevalence survey of hospital acquired infections at the hospital of the mother and child lagoon in Cotonou Benin Homel, March 2012

**DOI:** 10.1186/2047-2994-2-S1-P268

**Published:** 2013-06-20

**Authors:** G Batossi

**Affiliations:** 1 Hospital of Mother and Child Lagoon, Cotonou, Benin

## Introduction

Nosocomial infections (NI) pose a real public health problem because of their universal frequency, severity and socioeconomic consequences. While infection control is well organized in developed countries, it is less in countries with low economic level who suffer from a lack of regulations and of data monitoring. Prevalence surveys are the basic tool for the surveillance and prevention of NIs.

## Objectives

To measure the prevalence of NIs on a given day in order to determine HOMEL overall prevalence and specific infections.

## Methods

Our study was conducted at the Hospital of the mother and child Lagoon COTONOU on March 15, 2012. This is a descriptive cross-sectional survey measuring the prevalence of NIs "on any given day". All services were included in the survey except those where the stay did not exceed 48 hours. Coded data entry and analysis were performed by EPI-INFO.

## Results

105 patients were surveyed representing all patients present in the surveyed services for more than 48 hours at time of study. Our population was characterized by a slight female predominance. The sex ratio was 0 38. The average age was 15.02 years (SD with 19.45 years). The overall prevalence of Nis was 20.9%, and varied according to the sector of hospitalization. The ICU, Inpatient G and NICU were most affected with rates of 50%, 35.7%, and 24.1%, respectively. According to the site of infection, bacteremia / septicemia were the most frequent (50%). All infected patients were under antibiotic.

## Conclusion

This study shows that efforts are still needed to provide safe care to patients of the hospital in general. Areas for improvement are at the proper use of antibiotics, good sterilization and disinfection practices, and hand hygiene.

## Competing interests

None declared

